# Stabilized marker gene identification and functional annotation from single-cell transcriptomic data

**DOI:** 10.1371/journal.pcbi.1013574

**Published:** 2025-10-17

**Authors:** Sandesh Acharya, Pathum Kossinna, Qingrun Zhang, Jiami Guo

**Affiliations:** 1 Department of Cell Biology and Anatomy, University of Calgary, Calgary, Alberta, Canada; 2 Alberta Children’s Hospital Research Institute, University of Calgary, Calgary, Alberta, Canada; 3 Hotchkiss Brain Institute, University of Calgary, Calgary, Alberta, Canada; 4 Cumming School of Medicine, University of Calgary, Calgary, Alberta, Canada; 5 Department of Medical Science, University of Calgary, Calgary, Alberta, Canada; 6 Department of Biochemistry and Molecular Biology, University of Calgary, Calgary, Alberta, Canada; 7 Department of Mathematics and Statistics, University of Calgary, Calgary, Alberta, Canada; 8 Data Science Advisory Unit, University of Calgary, Calgary, Alberta, Canada; Georgia Institute of Technology College of Computing, UNITED STATES OF AMERICA

## Abstract

With the rapid emergence of single-cell transcriptomics datasets, reproducible marker genes and functional annotation of cell type or state is becoming increasingly important. Conventional methods that rely on differential gene expression (DEG) analysis lack both consistency across datasets and functional annotations of selected markers. Here, we present scSCOPE, an R-based platform that utilizes stabilized LASSO (Least Absolute Shrinkage and Selection Operator) feature selection, bootstrapped co-expression networks, and pathway enrichments to identify reproducible and functionally relevant marker genes and associated pathways in scRNAseq datasets. Using 9 scRNAseq datasets from human and mouse immune cells generated by different sequencing technologies, we show that scSCOPE outperforms other conventional methods by automatically identifying cell type-specific marker genes and pathways with the highest consistency across all datasets. scSCOPE’s gene co-expression and pathway analyses also provide in-depth molecular insights into the functionality of identified marker genes. We anticipate that scSCOPE will greatly improve cell type annotation and accelerate the design of experimental validation and functional investigations on cell heterogeneity.

## Author summary

With the growing number of single-cell transcriptomics datasets, it has become increasingly important to identify consistent markers that define cell types and their associated functions. However, existing methods rely heavily on analyzing individual gene expression, which can vary between experiments and fail to capture the bigger picture of how genes work together. In our study, we present **scSCOPE**, a computational tool designed to address these limitations. Unlike traditional methods, scSCOPE not only evaluates gene expression but also incorporates gene co-expression. This approach enables the identification of marker genes and pathways of cell types of interest with the highest consistency across all datasets and provides in-depth molecular insights into the functionality of identified marker genes. We anticipate that scSCOPE will greatly improve cell type annotation and accelerate the design of experimental validation and functional investigations on cell heterogeneity.

## Introduction

Single cell RNA sequencing (scRNAseq) enables high throughput profiling of transcriptomics for millions of cells at a time, and has transformed our understanding of cell heterogeneity, physiological state, and function in varied tissues across development and diseases [1–7]. Cell type identification in scRNAseq requires clustering of cells into distinct cell types based on their gene expression profiles, followed by the identification of marker genes associated with each cell type [8]. Currently, marker gene selection in scRNAseq data is an error-prone task. This is because common marker gene identification methods rely solely on differential gene expression (DEG) analysis, where the highest differentially expressed genes are often selected as cell-type specific markers [9]. Similarly, to infer cell-type specific functionality, current state-of-the-art methods use DEGs found in each cell type as inputs to look for pathway enrichments using databases such as KEGG, Gene Ontology [10–13]. These pathways are then ranked based on enrichment scores or p-values, which are directly influenced by the number of DEG inputs. Various state-of-the-art methods including Mast, Deseq2, Bimod, Wilcox Rank Sum Test, Roc, DESingle have been developed to identify DEGs in scRNAseq datasets [14–20]. These methods focus on mitigating the challenges associated with scRNAseq data’s inherent bimodality, dropout events, and technical variations [14–18,20,21]. However, there are two major limitations associated with these methods: (1) They analyze one gene at a time only based on expression values and do not consider gene-gene interactions, and therefore can be extremely sensitive to technical and biological variations in sample collection and sequencing platforms, resulting in low marker gene identification and pathway enrichment stability or consistency across datasets [22–24]. This is particularly problematic for rarer cell types or transient cell states that are not well characterized; (2) Secondly, these techniques lack the capability to functionally annotate each marker gene in a particular cell type. As a result, researchers must manually choose a few marker genes from the pool of DEGs for experimental validation, based solely on their differential expression, and without functional insights.

Genes do not operate alone. Hundreds of genes can be regulated by the same sets of transcriptional drivers. Incorporation of gene co-expression along with DEG analysis can help improve the identification of cell-type specific marker genes and pathways that represent functionally important molecular signatures of cell types/states that are stable across datasets. However, complex multi-gene models suffer from statistical instability, leading to inconsistencies when inputs are slightly altered [25–28]. This problem is particularly pronounced in scRNAseq analysis due to the variation associated with sequencing techniques and downstream data analysis [21].

To overcome these limitations, we have developed scSCOPE (single-cell Stabilized COre gene and Pathway Election), which utilizes stabilized LASSO (Least Absolute Shrinkage and Selection Operator) feature selection, bootstrapped co-expression networks, and pathways enrichments to identify stable and functionally relevant marker genes and associated pathways for cell type identification and functional annotation using scRNAseq datasets [27–32]. scSCOPE is an extension of SCOPE, our previously established bulk RNAseq analysis method that empowers the synergy between co-expression analysis and regularized multiple regressions to provide stable and robust predictions of marker genes and pathways [31].

We performed a systematic benchmarking of scSCOPE and other state-of-the-art methods [15–20] across 9 scRNAseq datasets including 6 human PBMC (Peripheral Blood Mononuclear Cell) [33] datasets and 3 mouse immune cell datasets generated by different sequencing technologies [34–36]. We also performed simulations to compare the performance of scSCOPE with other methods on synthetic datasets. Our results show that scSCOPE accurately identifies marker genes and pathways that (i) show a high degree of gene co-expression, an indicative of involvement in cell-type specific functional programs; (ii) represent cell-type specific molecular signatures that can be reliably captured regardless of technical variations across different scRNAseq datasets. Furthermore, scSCOPE automatically annotates each marker gene with enriched pathways and gene co-expression to facilitate cell-type specific functional annotations and validation.

## Results

### The framework of scSCOPE

**Fig 1:**
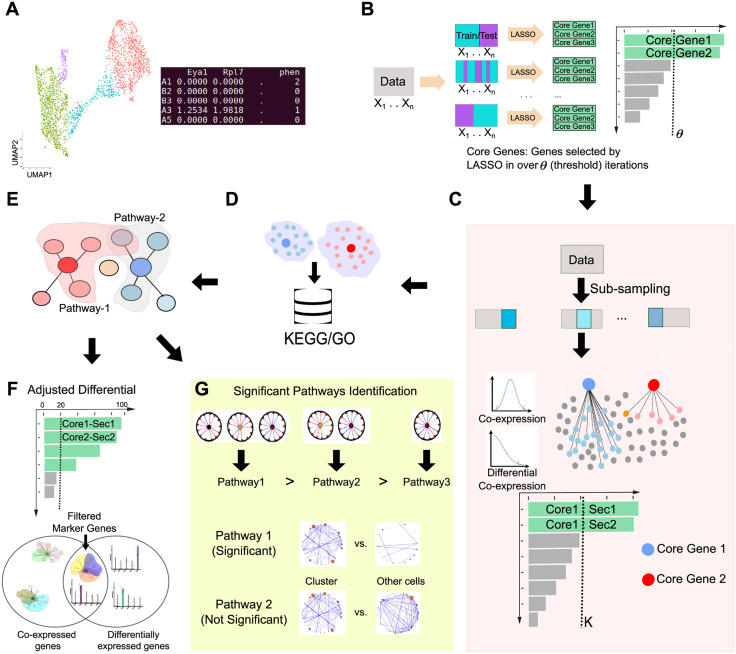
Overview of scSCOPE Framework. (A) scSCOPE requires a gene expression matrix along with cluster annotations as input. (B) The input data undergoes Iterative Sparse Lasso Regression to identify genes capable of segregating two distinct groups. In each iteration of LASSO regression, data is split into training and testing groups with the same cluster composition as in the original dataset. Only those genes consistently chosen in over a threshold (*θ*) of iterations are designated as Core Genes. (C) Core Genes are then subjected to Bootstrapped Co-expression analysis, where all the genes significantly co-expressed or differentially co-expressed with the core genes are identified as secondary genes. This analysis is run in a 60% sub-sample of the original data for 100 iterations and only stable gene interactions that appear in more than K iterations out of 100 are selected. (D) Each core gene, and its stable secondary genes identified through Co-expression analysis, then undergoes Pathway enrichment analysis. (E) Pathway analysis can identify not only pathways enriched in the cluster but also core-secondary gene interactions and their involvement across multiple pathways. (F, G) The results from co-expression analysis, differential expression, and pathway enrichment are analyzed together to determine marker genes and pathways for the cluster of interest. Each of these steps are repeated and run in parallel for each analysis.

The input for scSCOPE is a clustered single-cell RNAseq dataset with an expression matrix (Fig 1A). Based on scRNAseq data clustering (Fig 1A), scSCOPE begins by running a bootstrapped logistic LASSO (see Methods) to identify “core genes” that robustly separate two groups of cells in multiple iterations [30] (Fig 1B). These “core genes” then undergo bootstrapped co-expression network analysis (Methods) to identify their stably co-expressed genes (“secondary genes”) (Fig 1C) [28]. The “core-secondary” gene pairs are subsequently subjected to pathway enrichment analysis (Methods; Fig 1D), leading to a collection of pathways enriched in each cell type (Fig 1E) [32]. Next, the “core-secondary” gene pairs are ranked based on their pairwise correlations and enrichment in different pathways. Marker genes are then selected from all the genes in the top core-secondary gene pairs based on their differential expression (Methods, Fig 1F and 1G). All the marker genes identified by scSCOPE are annotated with the top pathways they are associated with. This provides important functional annotations of the selected marker genes (Methods). As a final step, scSCOPE employs a unique ranking system to assess the identified pathways by integrating the impact of both gene expression and co-expression (Methods). Taken together, scSCOPE harnesses information from multiple modalities to identify highly stable and functionally informative cell-type specific marker genes and pathways using scRNAseq data.

### scSCOPE identified marker genes that are stable across datasets and relevant to cell-type specific functions

To assess the accuracy, stability, and functional significance of the markers and pathways identified by scSCOPE, we collected 6 scRNAseq datasets on human PBMCs (Peripheral Blood Mononuclear Cells) generated from different sequencing technologies and 3 mouse immune cell datasets [33–36]. PBMCs, which include lymphocytes (T cells, B cells, and NK cells), monocytes, and dendritic cells, are one of the most well characterized cell types with identified cell markers and signaling drivers along each lineage [37]. The three mouse datasets are GSE109999, GSE168158 and Tabula Muris [34–36]. GSE109999 is a dataset of FACS-sorted immune cells (B-cells, T-cells, Granulocytes, Erythroblasts, and Progenitor cells) sequenced using CEL-seq2 technology [35]. As the cell types were determined prior to sequencing, this dataset is considered a gold-standard dataset (from here on referred to as “gsBlood” dataset). GSE168158 is a dataset of B-cells and its subtypes in the mouse bone marrow [36]. Tabula Muris is an extensive collection of single-cell transcriptome data obtained from around 100,000 cells representing 20 different organs in mice, including bone marrow (immune) cell types with pre-defined clusters that we analyzed here [34].

**Fig 2:**
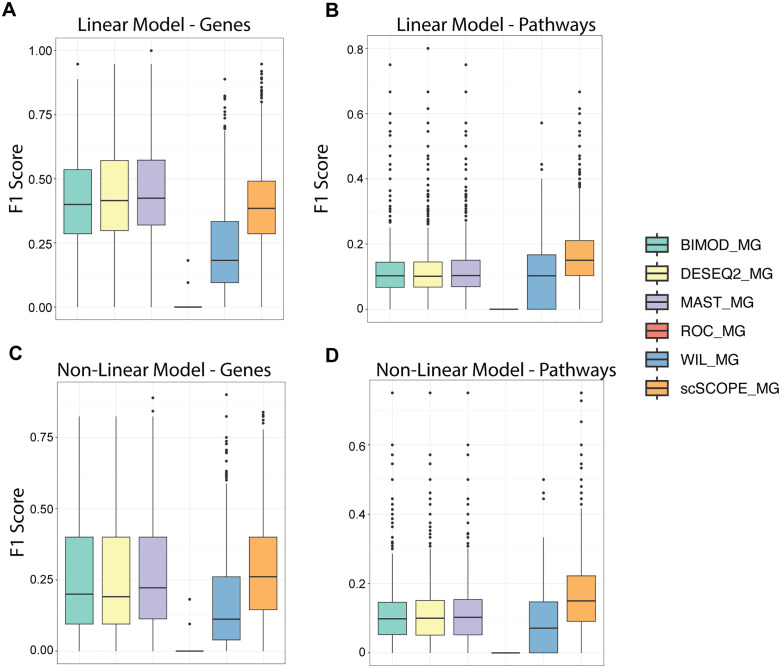
Simulations comparing the performance of scSCOPE with other methods. F1 score = {TP/[TP + 0.5 × (FP + FN)]} calculated for the accuracy of scSCOPE and other methods in identifying predictive genes and pathways simulated in the GTEX single-cell gene expression data using linear (A, B) and non-linear (C, D) models across all simulations.

To assess scSCOPE in comparison to alternative state-of-the-art methods including Deseq2, Wilcox, ROC, Bimod and MAST, we measured the performance of each method for marker gene identification in simulated and real datasets. Simulated datasets were generated from GTEX single-cell data using a set of highly correlated genes for phenotype prediction using linear and non-linear models [38] (Methods). For pathway identification, scSCOPE outperforms other methods across both linear and non-linear scenarios (Figs 2B, 2D and S1B). When a lower number of genes were available for classification, other methods showed better performance-likely likely due to the dataset’s lower complexity (S1A Fig). However, as the number of genes used for prediction increased, scSCOPE more accurately identified them as compared to other methods in both linear and non-linear models (S1A Fig). This improvement was particularly pronounced under nonlinear conditions, highlighting scSCOPE’s ability to detect complex gene-gene interactions compared to other conventional tools. Given that real scRNAseq data often exhibit unknown levels of correlation and may not conform strictly to linear assumptions, scSCOPE would likely outperform other conventional methods on marker gene identification. Thus, using simulated datasets, in comparison to other methods, scSCOPE demonstrated comparable performance under linear models and exhibited advantages under non-linear settings in identifying predictive genes (Figs 2A, 2C and S1A). In addition, we observed that scSCOPE exhibits a higher true positive rate (TPR) and a lower false discovery rate (FDR) in identifying both genes and pathways compared with other methods (S2 Fig). Across all simulation settings and methods, the number of false positive genes remained very low (fewer than five per simulation), whereas the number of false positive pathways was higher. This is because the same set of genes could be enriched in multiple overlapping pathways, which inflates pathway-level false discoveries.

**Fig 3:**
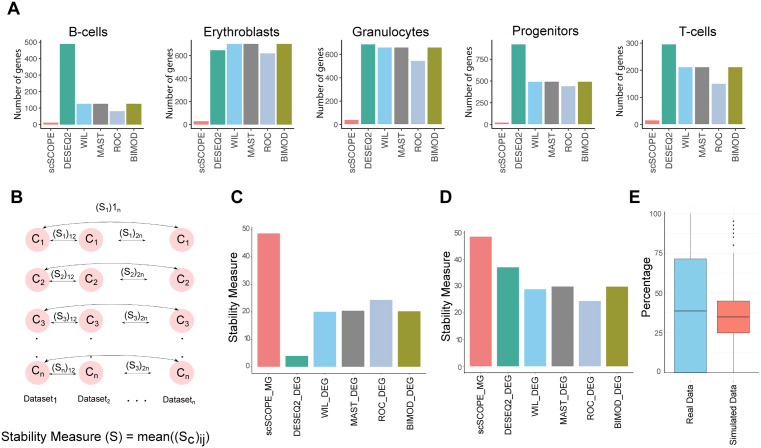
scSCOPE identifies fewer but more stable marker genes. (A) Bar Plots showing the number of marker genes identified by scSCOPE as compared to the DEGs identified by other methods. (B) The stability assessment of each method in identifying marker genes involves calculating the stability measure (S) based on the common marker genes detected within the same cluster across various datasets. This measure (S) is defined as the average of all (S_c_)_ij_ values, where (S_c_)_ij_ represents the percentage of common Marker Genes identified between dataset “i” and dataset “j” within cluster “c”. Here, “c” denotes the cluster of interest, “i” corresponds to dataset 1, and “j” corresponds to dataset 2. The (S_c_)_ij_ value is computed for all possible pairwise combinations of “i” and “j” for each cluster, and subsequently, these values are averaged to derive the overall stability measure (S). (C,D) Bar plots illustrating the stability measure of different methods for identifying marker genes in human PBMC datasets [33]. Top DEGs are selected based on their p-values in (C), and the average log fold change in (D). (E) Bar Plot showing the percentage of marker genes identified by Co-expression alone in Real and Simulated Datasets.

With real datasets, we noted that scSCOPE identified a small number of marker genes (Fig 3A) compared to all the DEGs provided by other methods (S1 Table). DEGs were filtered based on their adjusted p-value (<0.05) and abs(logFC) (> 0.25). To compare the stability of marker gene identification, we used 6 human PBMC datasets [33]. For DEGs identified by other methods, we chose the top genes ranked by the highest average log2 fold change or lowest p-values to compare with scSCOPE. The stability of the top DEGs identified by each method and scSCOPE markers were then tested across the different datasets for each cluster (Fig 3B). We found that scSCOPE markers showed the highest level of consistency across all 6 datasets compared to all other methods when selecting different number of top marker genes (5,10,20,50) ranked by both average log2 fold change and p-value. (Figs 3C, 3D, S3A, and S3B). We also observed that Correlated Gene Network identified by scSCOPE is more stable as compared to differentially expressed genes (S3C Fig). In addition, we observed that more than 25 percent of marker genes identified by scSCOPE are identified by Co-expression alone in both real and simulated datasets (Fig 3E). This suggests that scSCOPE’s incorporation of gene co-expression networks improves the stability of marker gene identification as compared to conventional methods that are based on single-gene differential expression analysis.

Since scSCOPE considers the level of gene co-expression as a criterion for marker gene selection, we next assessed gene co-expression levels of scSCOPE-markers among all the DEGs using the Wilcox rank sum test. We first identified DEGs for each cell type in the gsBlood dataset, which were then subjected to “hub-gene” analyses using the String database and Cytohubba plugin within the Cytoscape application [39–41]. “Hub genes” are genes with the highest number of co-expressed genes [25]. We observed that the top hub genes consistently aligned with the scSCOPE-markers (S4A to S4C Fig). For example, 10 out of the top 15 hub genes in B-cells, 8 out of the top 10 hub genes in T-cells, and 9 out of the top 10 hub genes in Granulocytes were identified as scSCOPE-markers. This high degree of convergence suggests that scSCOPE indeed automatically identifies markers that show high levels of gene co-expression. The hub genes not selected as markers by scSCOPE are denied using criteria including pathway enrichment and differential expression.

### scSCOPE provides functional annotations to cell-type specific markers

scSCOPE’s implementation of gene co-expression and pathway enrichment for marker identification automatically provides functional annotations to the selected markers. All the marker genes and their associated pathways are listed in S2 Table. The gene co-expression networks across multiple pathways can be visualized by gene network plots generated using the “geneNetwork” function from the R-implementation of scSCOPE. We also created an interactive interface for generating gene network plots accessible at Gene Network (https://sant7.shinyapps.io/geneNetwork/).

**Fig 4:**
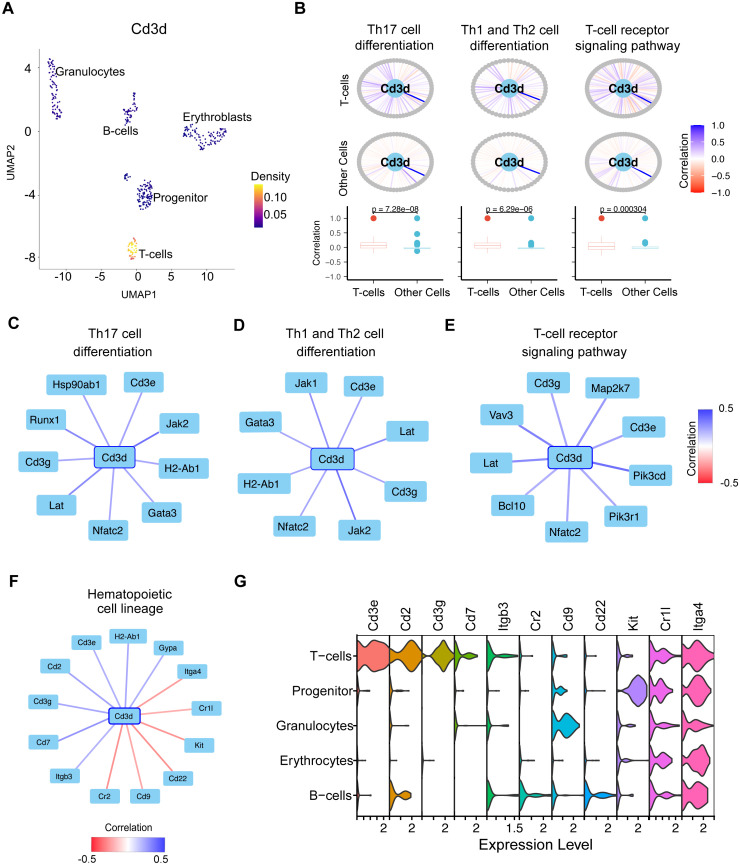
The role of *Cd3d* in T-cells as revealed by scSCOPE. (A) Density Plot showing the relative expression of *Cd3d* gene in different cell types in the gsBlood dataset. (B) The gene network plots for *Cd3d* gene provide a visual representation of its extensive interactions with genes across diverse pathways. Within each plot, surrounding *Cd3d* are gray circles representing all other genes within the pathway. The lines connecting *Cd3d* to these genes indicate Pearson’s correlation coefficient, ranging from -1 (red) to +1 (blue), reflecting the strength and direction of correlation. Correlations are separately calculated for two distinct groups: in this case T-cells and all other cell types. Boxplots accompanying the plots contrast the distributions of correlations between groups. *p*-values from the Kolmogorov-Smirnov test are provided to assess the statistical significance, with the null hypothesis stating that two samples are drawn from the same distribution. (C,D,E,F) Gene network plots are zoomed in to visualize only the top genes which show highest correlation (|Correlation| ≥ 0.2) with *Cd3d* across different pathways in T-cells. Lines are colored according to the Pearson’s correlation between two genes from -0.5 (red) to + 0.5(blue). (G) Violin Plots for genes significantly correlated with *Cd3d* in the Hematopoietic cell lineage pathway.

As an example, scSCOPE identified *Cd3d* as a marker for T-cells (Fig 4A) and automatically revealed that *Cd3d* exhibits significant co-expression with genes in T-cell specific pathways, including Th17 cell differentiation, Th1 and Th2 cell differentiation, and T-cell receptor signaling pathways (Fig 4B–4E). In addition, the gene network plot for *Cd3d* in hematopoietic cell lineage pathway reveals that *Cd3d* is positively co-expressed with genes specific to T-cell speciation and differentiation, including *Cd2, Cd7, Cd3g, Cd3e, Itgb3, H2-Ab1*, while negatively co-expressed with non-T-cell specific genes like *Cd9, Kit, Itga4, Cr1l, Cd22* and *Cr2* (Fig 4F and 4G). Thus, scSCOPE’s annotation is supported not only by the expression and co-expression patterns of *Cd3d*, but also by its enrichment within key T cell-related pathways. These findings align with the well-established functional role of *Cd3d* as a component of the T-cell Receptor (TCR)/CD3 complex, which is essential for T cell activation, differentiation, and the coordination of immune responses [42].

The gene network plot generated by scSCOPE is particularly useful to facilitate target gene prediction when a transcription factor is identified as a marker gene. For example, scSCOPE identified *Tcf7* (transcription factor 7), a gene encoding the transcription factor Tcf1 as a marker gene for T-cells in the gsBlood dataset (S5A Fig). In total, *Tcf7* gene exhibited significant co-expression (absolute value ≥ 0.2) with more than 1700 genes across the entire dataset. scSCOPE refined these gene co-expression to focus only on T-cell specific pathways, thereby isolating and prioritizing the most relevant gene interactions for T-cells. Gene network plots show that *Tcf7* is significantly co-expressed with 57 genes among T-cell related pathways (e.g., T-cell receptor signaling pathway, Th17 cell differentiation, Th1 and Th2 cell differentiation pathways) (S5B Fig). Notably, 49 out of these 57 genes have binding sites for Tcf1 in their promotor regions [43,44], and 38 out of these 49 genes have been validated using ChIPseq experiments [44] (S5C Fig). Of the remaining eight genes, four (*Maml2, Irf4, Hras, and H2-DMa*) are regulated by Runx1, a target gene of Tcf1 [43]. Tcf7’s strong connectivity within these immune pathways suggests a key regulatory role in T-cell development and function. Collectively, these results demonstrated that scSCOPE’s gene co-expression and pathway analyses provide enriched and in-depth molecular insights into the functionality of identified marker genes in each cell type.

### scSCOPE identified a new B-cell marker *Il7r*

scSCOPE utilizes a multi-step filtering process to pinpoint informative marker genes for cell type classification in scRNAseq data. Genes that can distinguish the cell-type of interest, show extensive co-expression with genes enriched in cell-type specific pathways, and that are differentially expressed are selected as markers in scSCOPE (S6A Fig). As a result, genes that rank low in DEG analysis but excel in co-expression and pathway analysis may be identified as markers by scSCOPE.

As an example, scSCOPE identified *Il7r*, a gene with a low differential expression score but substantial co-expression, as a marker gene for B-cells in the gsBlood dataset (S2 Table). Based on expression enrichment, Il7r ranks low among DEGs for B-cells (MAST: 46th, DESeq2: not identified as a DEG, Wilcox: 46th, ROC: 46th, Bimod: 46th) and is consequently not selected as a marker from previous scRNAseq studies (S6B Fig). However, when considering both the strength of gene co-expression and involvement in pathways, *Il7r* exhibits strong correlations with three core genes identified for B-cells (S6C Fig) and is enriched in two pathways specific to B-cell functions and is therefore identified as a B-cell marker by scSCOPE. In contrast, while genes such as *Cd74* and *Igkm* rank high in DEG analyses in B-cells, they do not exhibit significant co-expression or pathway enrichment and are consequently disregarded by scSCOPE as markers (S6B and S6C Fig).

Il7r is a cell membrane receptor for the interleukin-7 protein [45–48]. Extensive literature has reported the critical role of IL-7R signaling in the survival and maintenance of lymphocytes [45–52]. In the context of B-cells, IL-7R signaling has a well-defined role in promoting the proliferation and survival of B-cell progenitors [46,48–50,52]. To demonstrate the accuracy and depth of scSCOPE’s functional annotation of marker genes, we next evaluated the scSCOPE generated *Il7r* annotation alongside the known functions of IL-7R signaling in B-cells. We employed scSCOPE on the Tabula Muris dataset focusing specifically on the bone marrow and GSE168158 dataset. Tabula Muris dataset incorporates data from two experiments with distinct methodologies for cell isolation: droplet technology and FACS sorting. The droplet dataset offers broader coverage across B-cell stages, while the FACS dataset provides higher depth sequencing. The two datasets encompass diverse and partially overlapping developmental stages of B-cells. We leveraged both datasets to include all B-cell subtypes in our analysis.

The development of B-cells is dependent on the sequential DNA rearrangement of the immunoglobulin loci that encode subunits of the B cell receptor [48]. The hematopoietic progenitor cells undergo the B-cell lineage specification and commitment process from Pre-Pro, Pro-B (further divided to Early-Pro and Late-Pro), Pre-B, immature-B, to naïve B-cells [51,52]. Cell proliferation and survival, two major events during B-cell development, are both known to be regulated by IL-7R signaling, especially during the Pro-B and Pre-B stages [48–50]. Importantly, scSCOPE accurately identified *Il7r* as a marker specifically for Late-Pro and Pre-B stages.

**Fig 5:**
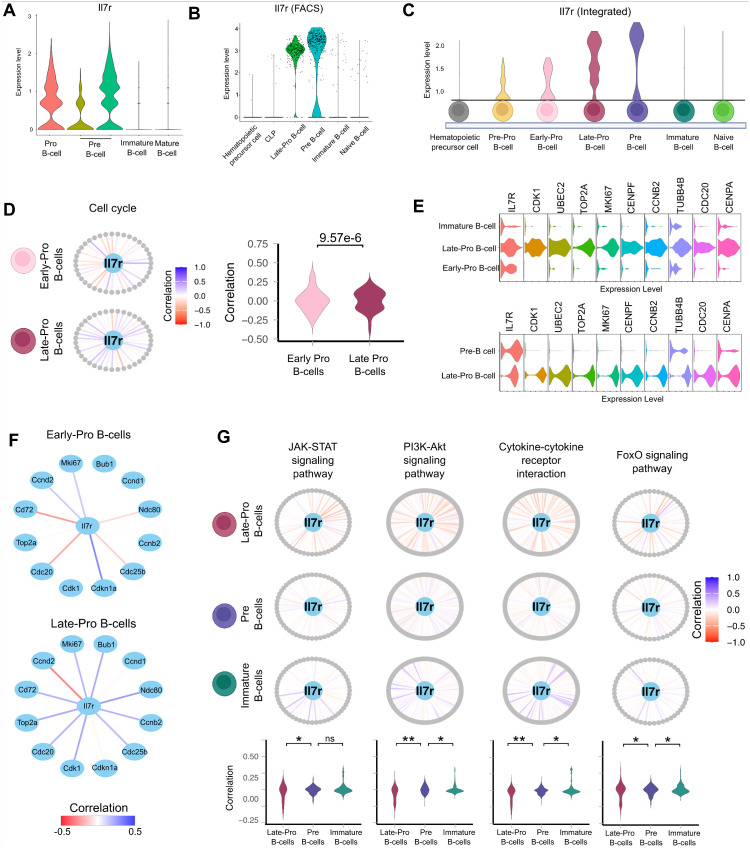
scSCOPE identified *Il7r* gene as a marker gene for Late-Pro and Pre-B cells. (A) Violin Plot showing the relative expression of Il7r gene across different B-cell stages in GSE168158 (B) Violin Plot showing the relative expression of *Il7r* gene across different B-cell stages in FACS dataset of Tabula Muris. (C) Relative expression of *Il7r* gene in different B-cell stages generated by integrating the FACS and Droplet datasets from Tabula Muris. (D) Gene network plots for *Il7r* in Cell Cycle Pathway in Early-Pro and Late-Pro B-cells of the Droplet-Tabula Muris dataset. *Il7r* gene is placed in the middle with all other genes expressed in the pathway placed in the periphery. The lines connecting *Il7r* to these genes indicate Pearson’s correlation coefficient, ranging from -1 (red) to +1 (blue), reflecting the strength and direction of correlation. Correlations are separately calculated for Early-Pro B-cells and Late-Pro B-cells. Violin Plots accompanying the plots contrast the distributions of correlations between these groups. *p*-value from the Kolmogorov-Smirnov test is provided to assess the statistical significance, with the null hypothesis stating that two samples are drawn from the same distribution. (E) Violin Plots show the relative expression of G2M-phase genes in different clusters in the FACS and Droplet Tabula Muris datasets. (F) Correlation Plots for *Il7r* with selected genes in the Cell Cycle Pathway across Early-Pro and Late-Pro B-cells. Lines are colored according to the Pearson’s correlation between two genes from -0.5 (red) to + 0.5(blue). (G) Gene network plots for *Il7r* gene in Late-Pro, Pre-B and Immature B-cells of the FACS-Tabula Muris dataset. Correlations are separately calculated for three groups. Violin Plots accompanying the plots contrast the distributions of correlations between these groups. P-values (Kolmogorov-Smirnov test) are denoted: *, p < 0.05; **, p < 0.01; ***, p < 0.0001; ns, p ≥ 0.05. Corresponding p-values for Late-Pro *vs.* Pre-B cells are: 0.0023 (JAK-STAT), 0.00013 (PI3K-Akt), 0.00034 (Cytokine-cytokine), 0.010 (FoxO). Corresponding p-values for Pre-B *vs.* Immature B-cells are: 0.054 (JAK-STAT), 0.024 (PI3K-Akt), 0.047 (Cytokine-cytokine), 0.016 (FoxO).

Expression wise, consistent with its marker status, *Il7r* indeed shows the highest expression levels at the Late-Pro and Pre-B stages compared to all other stages (Fig 5A, 5B, and 5C). scSCOPE’s gene network plots highlighted that *Il7r* is significantly co-expressed with genes enriched in cell cycle and survival-related pathways (Fig 5D and 5G). For cell cycle regulation, scSCOPE revealed that *Il7r* shows an increased co-expression correlation in the cell cycle pathway at Late-Pro compared to Early-Pro stage (Fig 5D). Indeed, *Il7r* is positively co-expressed with many cell-cycle related genes like *Bub1*, *Mki67*, *Cd72*, *Cdc20*, *Ndc80*, *Top2a*, *Cdc25b* that promote B-cell proliferation in the Late-Pro stage compared to Early-Pro and Pre-B stages (Fig 5D and 5F). For cell survival regulation, IL-7R signaling is known to activate downstream STAT5 transcription factor and PI3K-AKT signaling to promote cell survival along B-cell development [48]. Consistently, scSCOPE’s co-expression and pathway enrichment analyses revealed that *Il7r* is co-expressed with genes enriched in JAK-STAT, PI3K-AKT, and related pathways such as the FoxO pathway that is repressed during cell survival, and the Cytokine-Cytokine receptor interaction pathway important for B-cell differentiation (Fig 5G). Interestingly, from the Late-Pro to Immature B cell transition, *Il7r*’s co-expression patterns in these pathways show a shift from a predominantly negative correlation to positive correlation (Fig 5G), indicating that IL-7R signaling activity is dynamically regulated during this process, consistent with experimental findings [48]. Based on these results, we predict that IL-7R signaling plays a critical role in promoting proliferation during the Pro-B cell stage and in facilitating the developmental transition from Pro-B to Pre-B and Immature B cells. Although *Il7r* gene expression or co-expression correlation is not a direct readout of IL-7R signaling activity, these analyses demonstrate scSCOPE’s great sensitivity and accuracy in providing insights into the dynamic regulation of *Il7r* and related signaling pathways during early B-cell differentiation.

### scSCOPE enabled functional prediction of the unannotated gene Gm8292

**Fig 6:**
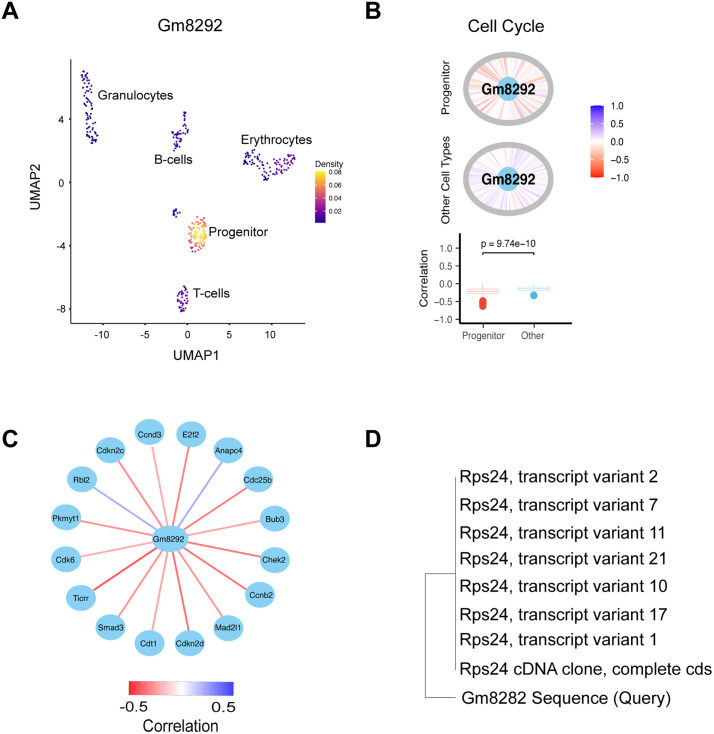
scSCOPE’s functional annotation of an un-annotated gene *Gm8292.* (A) Density Plot showing the expression level of *Gm8292* in different clusters of gsBlood dataset. (B) Gene Network Plot for *Gm8282* shows that this gene is co-expressed with many genes within the Cell Cycle pathway. All the genes in Cell Cycle pathway are placed in the circumference of the circle (Grey color) and *Gm8292* gene is placed in the center (Light blue). Lines represent the strength and direction of Pearson’s correlations between *Gm8292* and all other genes from -1 (red) to +1 (blue). Boxplots contrast the distributions of two sets of correlation in the Progenitor cells vs all other cell types. The Kolmogorov-Smirnov test was performed, with the null hypothesis being that two samples are drawn from the same distribution. (C) Gene network plots showing correlations between *Gm8292* and selected genes from the Cell Cycle Pathway (with absolute correlation > 0.2). (D) Neighbor-Joining phylogenetic tree depicting the sequence similarity of the Gm8292 gene to entries in the *Mus musculus* non-redundant (nr) nucleotide database, based on BLAST analysis.

In cases where scSCOPE identifies unannotated or poorly characterized genes as markers, their gene network plots can offer valuable clues about their potential functions within the cell type of interest. As an example, scSCOPE identified *Gm8292* as a marker gene for hemopoietic progenitor cells in the gsBlood dataset (Fig 6A). *Gm8292* (ENSMUSG00000100215) is a mouse pseudo-gene on chromosome 1. Its expression has been associated with conditions such as cholestatic intestinal injury and TCDD-induced cleft palate [53,54]. However, its function remains unknown. scSCOPE’s Gene Network Plot of *Gm8292* revealed its significant correlation with multiple genes in the “Cell Cycle” pathway (Fig 6B). Interestingly, *Gm8292* showed a negative correlation with many genes involved in “Cell cycle arrest” including *Ticcr*, *Cdkn2d*, *Chek2*, *Mad2l1*, *E2f2*, *Cdkn2c*, and *Pkmyt1* (Fig 6C). Thus, Gm8292 could be involved in cell cycle regulation and likely plays a role in the promoting cell cycle progression important for hemopoietic progenitor cells proliferation and self-renewal.

To further investigate its identity and potential function, we extracted the Gm8292 genomic sequence from chromosome 1 and performed a BLAST search against the *Mus musculus* non-redundant (nr) nucleotide database [55,56]. Remarkably, Gm8292 displayed high sequence similarity to several alternative transcripts of the Rps24 gene (Fig 6D), a known component of the ribosomal protein family. A review of the literature reveals that Rps24-deficient cells exhibit elevated levels of the cell cycle inhibitor p21, alongside upregulation of Cyclin E, Cdk4, and Cdk6 [57,58]. These findings align with our co-expression analysis and support the hypothesis that Gm8292 may function analogously to Rps24, potentially contributing to cell cycle progression. Importantly, although the sequence homology between Gm8292 and Rps24 was not initially known, the gene’s putative function could be reasonably inferred based on its co-expression network. This underscores the utility of scSCOPE in functional prediction of unannotated genes.

### scSCOPE identified more cell-type enriched pathways and more genes within each pathway

As many genes are co-regulated within the same signaling pathways, incorporation of co-expressed genes will help identify more cell-type specific pathways. However, conventional co-expression analysis with an extensive list of DEGs can significantly escalate computational complexity and time. scSCOPE’s pathway enrichment method uses both core genes (identified by bootstrapped LASSO) and secondary genes (co-expressed with core genes) as inputs to identify enriched pathways in each cluster of interest. Compared to common pathway analyses such as GSEA and ORA [32,59] that use DEGs to find enriched pathways, scSCOPE excelled by identifying (i) a higher number of enriched pathways, (ii) more stable pathways across different datasets, and (iii) more number of genes within each pathway (S7A, S7B, and S8 Figs and S1, S3 and S4 Tables). For example, in the gsBlood dataset, pathways such as the “IL-17 signaling pathway” which is important for granulocytes development was not identified by ORA or GSEA for Granulocytes but was identified by scSCOPE. This expanded pathways and genes per pathway not only substantiates the robustness of pathway enrichment analysis but also provides researchers with a more comprehensive list of potential regulators and effectors.

To highlight the relevance of enriched pathways in each cell type, scSCOPE employs a unique ranking system to indicate the significance of the identified pathways. scSCOPE calculates the weights of each pathway to generate “CorrExpress” values (Methods) by incorporating the correlation and expression differences of all genes within a pathway (Fig 1A, Methods). The top pathways for each analysis are then ranked based on the absolute values of CorrExpress, referred to as “Final Measure”. As a result, scSCOPE outputs a Pathway Bar Plot to identify the top pathways relevant for a cell-type as well as pathway network plots to study the patterns of expression and co-expression of genes in the pathway in different cell types (S7C and S7D Fig). Pathway network plots can be generated using the “pathwayNetwork” function and pathway bar plots can be generated using the “pathwayBar” function from R-implementation of scSCOPE. Additionally, these plots can be also created by using an interactive web interface accessible at Pathway Network (https://sant7.shinyapps.io/pathwayNetwork/).

### High biological significance of scSCOPE comes at an expense of time complexity

scSCOPE is substantially slower than current state-of-the-art differential expression tools due to its reliance on extensive bootstrapping (S9A Fig). For example, identifying marker genes in a dataset of 100,000 cells can take up to hours, whereas conventional tools complete the task in under a minute (S9A Fig). A notable advantage, however, is that scSCOPE generates intermediate output files, enabling the analysis to resume from the last completed step rather than restarting entirely, which is an especially useful feature for long-running jobs. Compared to its bulk-cell counterpart (SCOPE), the sparse implementation of lasso regression in scSCOPE is four fold faster (S9B Fig). In the case of correlation estimation, scSCOPE and SCOPE exhibit similar runtimes when the number of features is small, but as feature dimensionality increases, sparse implementation scSCOPE scales more efficiently and ultimately outperforms SCOPE in computational speed (S9C Fig).

## Discussion

Here we present scSCOPE, an optimised toolbox for single-cell RNA-seq based cell-type identification and functional annotation. To the best of our knowledge, scSCOPE is currently the only computational tool that implements gene co-expression, pathway enrichment and differential expression to identify marker genes in single cell transcriptomics data. scSCOPE is also the first tool to use genes co-expression to identify and rank pathways in single-cell transcriptomics data. To promote its application, scSCOPE is implemented as an open-source R-implemented tool (https://github.com/QingrunZhangLab/scSCOPE) to enable fully automated scRNAseq based cell-type functional annotation.

In comparison with other computational approaches that require manual inference for marker gene selection and pathway enrichment analysis based on differential expression, scSCOPE enables automatic identification of markers that are not only cell-type specifically enriched but also highly interactive in cell type-specific pathways in an unsupervised manner. Using 9 scRNAseq datasets of well-characterized immune cell types in humans and mice by different sequencing technologies (i.e., SMART-seq2, 10X_v2, 10X_v3, Dropseq, CelSeq, inDrops), we benchmarked scSCOPE against other state-of-the-art methods (DESeq2, Wilcox Rank Sum, MAST, ROC, Bimod). Overall, our results demonstrated that scSCOPE (i) showed the highest degree of stability in cell type-specific marker gene and pathway identification across all datasets; (ii) identified not only the well-established marker genes but also new marker genes based on their extensive gene co-expression within the cell type-specific pathways, despite their relatively low expression enrichment; (iii) enabled the functional prediction of an unannotated marker gene, and (iv) lastly, identified more cell-type specific pathways and more enriched genes within each pathway. We anticipate that with these powerful advancements, scSCOPE will greatly improve cell type/state annotation and accelerate the design of experimental validation and functional investigations on cell diversity, particularly when it comes to rare cell types or transient cell states that are poorly characterized, highly dynamic, and sensitive to external stimuli.

As an example, we demonstrated that scSCOPE identified *Il7r* as an important marker gene for Late-Pro and Pre-B cells in different independent datasets. Extensive experimental research [45,48,60–62] has established *Il7r* as a well-known key gene for B-cell differentiation. Nevertheless, due to its relatively low differential expression score, no scRNAseq analysis methods, except for scSCOPE, identified *Il7r* as a marker gene. Importantly, while genes such as *Il7r* that encode signaling molecules (e.g., cell surface receptors, transcription factors) can have powerful impacts on cell differentiation or cell state transitions, they often do not express at high levels and therefore are rarely identified as markers in scRNAseq datasets. The fact that scSCOPE selects *Il7r* as a marker gene highlights its unique ability to identify functionally key genes as cell type markers. Notably, Late-Pro and Pre-B cells represent transient cell states, transitioning from progenitors to immature B-cells. The scSCOPE’s identification of *Il7r* as a marker during these states highlights its high sensitivity and accuracy in capturing the gene signatures that potentially drive cell lineage/state transitions.

For pathway enrichment, scSCOPE identifies more pathways than other methods. Incorporation of co-expressed genes during pathway enrichment also adds a layer of consistency across different datasets. In addition, scSCOPE introduces a novel validation strategy for inferred pathways, based on the expression and co-expression patterns of genes. This innovative approach adds a layer of rigor to the analysis, improving the distinction between biologically meaningful pathways and potential artifacts. When many genes in a pathway exhibit both differential expression and differential co-expression in the cell type of interest, it indicates a potential significance of the pathway in driving cell-type specific biology.

scSCOPE has several limitations. First, since the input for scSCOPE is a gene expression matrix of all the cells and the phenotype/cluster annotation for each cell, one limitation of scSCOPE is that it relies on accurate clustering. If the initial clustering is wrong, scSCOPE fails to identify unique marker genes and pathways for the cluster of interest. Second, scSCOPE offers rich biological insights at the cost of computational runtime because of its extensive bootstrapping. This limitation is mitigated by intermediate output files, allowing a stoppable multi-step analysis process rather than an all-or-none process. Lastly, the gene network analysis in scSCOPE heavily relies on good sequencing depth and coverage of the transcriptome. With continuing advancements in single-cell sequencing technologies, the accuracy and sensitivity of scSCOPE is expected to increase accordingly.

In conclusion, scSCOPE’s consideration of gene networks, novel pathway validation strategy, and comprehensive pathway enrichment analysis not only enhance our ability to identify critical genes and pathways specific to each cell type but also offer more biologically meaningful perspectives on cellular heterogeneity.

## Materials and methods

### scSCOPE framework

The SCOPE framework was designed to identify candidate genes and pathways separating normal and diseased tissues in bulk RNA-seq datasets. We have further improved the SCOPE framework to be applied directly to single cell datasets to identify cell-type specific marker genes and pathways.

### SCOPE-stabilized LASSO selection

The initial step in scSCOPE involves the deployment of the LASSO algorithm to discern core genes capable of distinguishing between two groups [27]. Addressing the inherent instability associated with the LASSO algorithm, the SCOPE methodology uses bootstrapped LASSO regression to select genes exhibiting consistent behavior across multiple iterations [31,63,64]. However, the SCOPE methodology, which was developed for binary phenotypes and bulk RNA-seq data, could not be applied directly to single-cell data with multiple phenotypes. We therefore introduced “1 vs all” and “1 vs 1” logistic regression models to identify core genes associated with each cluster and differentiating between two clusters, respectively, in scSCOPE. Consistent with SCOPE’s methodology, LASSO regression iterates 200 times on sub-sampled data (split 70–30) and genes selected in over θ runs, termed Core Genes, are chosen for subsequent analysis. The default value for θ is 160 (80% of total iterations). In cases where no core genes are identified, the algorithm automatically selects the top five genes which appear most frequently in the LASSO iterations as core genes. To address the sparsity inherent in single-cell data, we used sparse matrices during LASSO regression.

### Co-expression and pathway analysis

Genes function within complex networks, interacting with other genes across various pathways to shape specific phenotypic traits [1,2,22]. Despite the good predictive performance of LASSO, it suffers from unstable selections of correlated variables and inconsistent selections of linearly dependent variables [31,63,64]. The original SCOPE framework utilizes Co-expression and Differential Co-expression analyses to reveal genes that are strongly associated with each core gene, which could be missed by LASSO feature selection [31].

To quantitatively analyze these relationships, we computed pairwise correlations between core genes and all other genes using the corSparse function from the qlcMatrix package [65], which accommodates for sparse matrices in single-cell data. Only those gene pairs that surpassed predefined thresholds for either differential co-expression or co-expression were deemed significant. These thresholds were meticulously determined. For correlation, we extracted the 97.5th percentile from a null distribution of correlations calculated among 1000 random genes in 100 rounds. We repeated this for both positive and negative threshold calculations. Similarly, for differential co-expression, we identified the 97.5th percentile from a null distribution of differential co-expressions (Correlation_group1_ – Correlation_group2_) between 1000 random genes across 100 rounds. Importantly, these threshold values were established separately for each cluster or analysis.

Only those genes demonstrating either pronounced co-expression or significant differential co-expression with the core genes, called secondary genes, were advanced for further analysis. To ascertain the stability of core-gene/secondary-gene correlations, we performed 100 bootstraps of co-expression analysis on a sub-sample, representing 60% of the entire dataset. Sampling was done on each iteration. This sub-sample preserved the original dataset’s cluster distribution. Only core-secondary gene pairs deemed significant in over K sub-sampled rounds were retained for further analysis. The default value for K was 80.

For each core gene and its associated secondary genes, we conducted an Over-Representation Analysis (ORA) using the KEGG Pathway and Gene Ontology Database [10–12]. This analysis, executed through the WebGestaltR platform, applied a stringent false discovery rate (FDR) threshold of 0.05 to highlight pathways of notable significance [13].

### Marker gene identification

After identifying gene pairs that exhibit notable correlation or differential co-expression between core and secondary genes, we introduced a metric known as “adjusted differential correlation” to effectively rank these pairs.

${\text{Adjusted Differential}}_{\text{ij}}\;=\;\frac{{path}_{ij}}{\text{max}\left({path}_{ij}\right)for\;all\;i,j\;pairs}\;*\;\frac{{diffmet}_{ij}}{{\text{max}(diffmet}_{ij}for\;all\;i,j\;pairs}$, where

*i* = core gene, *j* = secondary gene, *path*_*ij*_ = number of pathways the gene pair (*i,j*) is involved in, ${diffmet}_{ij}\;=\text{ max}({correlation}_{ij},\;{group\text{1}\_correlation}_{ij},\;{group\text{2}\_correlation}_{ij},\text{ d}{differential\_correlation}_{ij})$ is the maximum of correlation of the two genes or differential correlation of the two genes between two groups.

The computation of adjusted differential correlation involves two components: the path ratio and diffmet ratio. The path ratio is the ratio of the number of pathways the core-secondary pair is enriched in to the maximum number of pathways any core-secondary pair is enriched in. Similarly, diffmet ratio is the ratio of diffmet values for the core-secondary pair to the maximum value of diffmet for the cluster. Thus, the adjusted differential metric considers both the degree of differential co-expression/correlation, and the pathways associated with the gene pairs. In simpler terms, gene pairs that are linked to multiple pathways and display substantial correlation or differential co-expression hold more significance as compared to others.

To refine our focus, we established a threshold for adjusted differential correlation. Only gene pairs surpassing 20% of the maximum adjusted differential correlation within the cluster were deemed significant. In cases where the counts of identified marker genes are exceptionally low, this threshold can be relaxed to capture additional genes of interest.

Finally, we evaluated the fold change for each gene from the above pairs. Marker genes were then filtered using a threshold for both fold change (0.5) and the fraction of cells expressing the gene (0.45). This stringent criterion helped to pinpoint genes that play a substantial role in characterizing the specific cluster under analysis.

### Stability calculation

We used human blood cell datasets from different platforms (10x-v3, 10x-v2, Dropseq, CELSeq, Seqwell and inDrops) generated by Ding et. al [33] to test for the stability in DEG and pathways identified by different methods. To do this, we identified significant DEGs (adjusted p-value < 0.05) in each cluster of all the datasets using different methods (Wilcox Rank Sum, Bimodal analysis, MAST, DESEQ2, and ROC) [15–18,20]. We selected the top 50 DEGs (ranked by either their absolute average fold change or by their p-values) as marker genes from different methods. Next, we calculated overlaps between DEGs in each cluster of one dataset with the same cluster from another dataset. Pairwise overlaps over all clusters and all datasets were averaged to generate a new value “Stability Measure”. Similarly, we also calculated the Stability measure for marker genes identified by scSCOPE. We also calculated stability measures for the pathways identified by scSCOPE and other methods in the same datasets.

### Stable pathways identification

We performed pathway enrichment for each core gene and its surrounding genes for each cluster separately. For each identified pathway, we calculated the number of core-secondary gene pairs enriched in the pathway. Next, we calculated the CorrExpress measure to differentiate between the expression and correlation pattern in the cluster of interest and all other cells. CorrExpress measure was calculated separately for genes expressing positive differences (posCorrExpress) between two clusters and genes expressing negative differences (negCorrExpress) between two clusters.

For genes i with$\;\left\{\;\;\;{\left({exp}_{i}\right)}_{cluster}-{\left({exp}_{i}\right)}_{othercells}\right\}\geq \text{0}:$

$\text{posCorrExpress }=\;\frac{\sum_{i}{\left({exp}_{i}\right)}_{cluster}\;-{\left({exp}_{i}\right)}_{othercells}}{n_{i}}\;*\;\frac{\sum_{i,j}abs|{{(cor}_{ij})}_{cluster\;}-\;{{(cor}_{ij})}_{othercells\;|}}{n_{ij}}\;$

and for genes i with $\;\left\{\;\;\;{\left({exp}_{i}\right)}_{cluster}-{\left({exp}_{i}\right)}_{othercells}\right\}<\text{0}:\;$


$$\text{negCorrExpress }=\;\frac{\sum_{i}{\left({exp}_{i}\right)}_{cluster}\;-{\left({exp}_{i}\right)}_{othercells}}{n_{i}}\;*\;\frac{\sum_{i,j}abs|{{(cor}_{ij})}_{cluster\;}-\;{{(cor}_{ij})}_{othercells\;|}}{n_{ij}}$$

where $\sum_{i}$ denotes the sum over all genes in the pathway, ${\left({exp}_{i}\right)}_{group\text{1}}$ represents the average expression of gene i in the first group, ${\left({exp}_{i}\right)}_{group\text{2}}$ represents the average expression of gene i in the second group, and $n_{i}$ is the total number of genes in the pathway. Similarly, $\sum_{i,j}$ denotes the sum over all pairs of genes in the pathway, ${\left({cor}_{ij}\right)}_{group\text{1}}$ represents the Pearson’s correlation between genes i and j in the first group, ${\left({cor}_{ij}\right)}_{group\text{2}}$ represents the Pearson’s correlation between genes i and j in the second group, and $n_{i},j$ is the total number of gene pairs in the pathway.

The calculation of the pathway difference metric involves two key steps. First, the differences in the expression levels of individual genes within the specified pathway between two groups are added together and normalized. This is done separately for genes expressing positive differences as well as genes expressing negative differences between two groups. Second, the absolute differences in pairwise correlations among all gene pairs across the two groups are calculated and averaged. These two resulting values are multiplied, creating a composite metric, “Final Measure”, that effectively combines the influences of both gene expression variations and correlation dynamics within the pathway. Finally, top pathways for each analysis are ranked based on the absolute values of posCorrExpress or negCorrExpress metrics. Moreover, scSCOPE extends its analysis by attributing significance levels to both expression and co-expression differences between two groups. This is achieved by randomly selecting an equivalent number of genes as those in the pathway and computing the expression and co-expression differences between the two groups. The resulting distributions for expression and co-expression differences from these random genes are then compared to those of genes from the pathway. This comparison is carried out using a Kolmogorov-Smirnov Test (K-S test), yielding separate p-values for both expression and co-expression differences [66]. The correlation and expression difference between two groups in each pathway can be visualized using pathway network plots generated using visNetwork library in R [67].

By integrating both gene expression and correlation aspects, the pathway difference metric offered a thorough evaluation of the pathway’s significance. This comprehensive assessment facilitated the prioritization and ranking of relevant pathways within each cluster.

### Accounting for uneven datasets

Correlation analysis can be highly affected by the number of observations in each cluster of interest. We have incorporated a maximum sampling strategy to account for this problem. A threshold for maximum number of samples in each phenotype is used such that all different clusters have similar number of cells. This is implemented on each iteration of Logistic LASSO and Correlation analysis.

### Pathway annotation for each marker gene and gene network plots

To annotate each marker gene with pathways, we counted the number of genes in the pathway which were significantly correlated with our marker gene of interest. Those pathways with a higher degree of association with the marker gene were determined to be more significant for the marker gene in the cluster of interest. Gene network plots were generated based on Pearson’s correlation values between the marker gene and all other genes in the pathway across two groups. To look at the significance of correlation difference between two groups, the KS test was performed between pairwise correlations of group one with group two and was represented by a box plot/violin plot in the gene network plots. In cases where there were fewer than 100 gene-gene correlation values, sampling with replacement was carried out to take at least 100 correlation values for analysis.

### Hub gene identification using Cytoscape

A list of differentially expressed genes were input into the Cytoscape application and the full STRING network for the list was generated using the STRING database [39–41]. Next, we calculated node scores for each gene using the Cytohubba plugin and ranked them based on their degrees (interaction). The top 15 nodes/genes were selected as hub genes.

### Data generation and model fitting for simulations

Simulated datasets were generated by systematically varying key parameters, including signal-to-noise ratio (SNR), phenotype architecture (linear vs. nonlinear), and gene co-expression structures. Single-cell gene expression data from the GTEx project served as the input source [38]. For each simulation, a subset of core pathways was selected (p = 5,10,15), and genes (n = 10, 20, 30, 40) were prioritized based on their normalized absolute summed Pearson correlation with other genes within the selected pathways and their prevalence across multiple pathways. This strategy ensured the selection of genes with both high connectivity and pathway relevance. Additional genes were randomly sampled to yield a total of 2,000 genes per dataset.

The effect sizes (β) for selected genes were drawn from a uniform distribution: β∼Unif([−10,−5]∪ [5,10 ]). For the remaining additional genes, β values were sampled from a lower range: *β* ∼ Unif([−0.1,0.1]). Phenotypes were then simulated using either linear or nonlinear models. To ensure target SNR levels (0.7, 0.8, 0.9), Gaussian noise was added to the phenotype signal, followed by logistic transformation to obtain binary outcomes. Each unique parameter combination was repeated 50 times, yielding a total of 3,600 simulations. For nonlinear models, only pre-defined interaction terms among the selected genes were incorporated into the model to preserve statistical power. These interaction structures were made available across all evaluated methods to ensure a fair and consistent basis for comparison.

### Implementation in real datasets

We implemented scSCOPE in nine immune cell datasets to validate the accuracy and applicability. Immune cell datasets were chosen because they were highly annotated as compared to other cell types. We implemented both “1 vs 1” and “1 vs all” logistic regression to identify marker genes in each cluster as well as between two clusters in this study. The datasets used in this study are:

### GSE109999: A gold-standard immune cell dataset

This dataset consisted of FACS-sorted single cells representing B-cells, granulocytes, erythroblasts, and progenitor cells sorted from bone marrow, and T-cells isolated from the Thymus of 10–13 weeks old female C57/BL6 mice [35]. These isolated cells were subsequently pooled together and subjected to sequencing using the CEL-seq2 protocol. Since the biological cell types were FACS-sorted prior to sequencing, this dataset can be labeled as a gold-standard reference for cell type identity.

### Tabula Muris

Tabula Muris, a multifaceted compendium of single-cell transcriptome data derived from the model organism *Mus musculus*, comprises nearly 100,000 cells from 20 distinct organs and tissues [34]. In our study, we used a subset of the Tabula Muris dataset, specifically originating from the bone marrow. The Tabula Muris dataset comprises two methods for transcriptomic analysis: One utilizes microfluidic droplet-based 3’-end counting, which surveys thousands of cells per organ with relatively low coverage (Droplet). The other employs FACS-based full-length transcript analysis, providing higher sensitivity and coverage for more detailed insights from fewer cells (FACS).

### GSE168158

This dataset integrates single-cell RNA sequencing and CITE-Seq proteomics to profile 7,454 bone marrow-derived B cells from two wildtype C57BL/6 mice, capturing multiple transcriptionally distinct clusters spanning early B-cell development stages [36].

### Human datasets

To assess the stability of scSCOPE and other DGE methods, we used PBMC human datasets generated by Ding et. al in the same tissue by using multiple methods, including Dropseq, 10x V2, 10x V3, inDrops, Cel-Seq and Seqwell [33].

### Marker gene identification using other methods

Differential expression tests for Wilcox Rank Sum’s test, MAST, DESeq2, Bimod and ROC were carried out using the FindMarkers() function from Seurat [20]. An average log fold change cut-off of 0.5 was used and the marker genes not expressed by at least 45% of cells in any group were discarded.

## Supporting information

S1 Fig Simulations comparing the performance of scSCOPE with other methods. F1 score ={TP/[TP + 0.5 × (FP + FN)]} calculated for the accuracy of scSCOPE and other methods in identifying predictive genes (A) and pathways (B) simulated in the GTEX single-cell gene expression data using linear and non-linear models under different combinations of number of predictive genes and pathways.
(TIF)

S2 Fig Simulations comparing the performance of scSCOPE with other methods. True Positive Rate (TPR) and False Discovery Rate (FDR) calculated for scSCOPE and other methods in identifying predictive genes (A, B) and pathways (C,D) simulated in the GTEX single-cell gene expression data using linear and non-linear models under different combinations of number of predictive genes and pathways respectively.
(TIF)

S3 Fig scSCOPE identified marker genes are more stable as compared to DEGs. Bar plots illustrating the stability measure of different methods for identifying marker genes in human PBMC datasets [33]. Top DEGs are selected based on their average log fold change in (A), and p-values in (B). (C) Bar plots showing the stability comparison of scSCOPE identified Correlated Gene Network with top genes based on average fold change identified by other methods.
(TIF)

S4 Fig Correlation of Hub Genes and scSCOPE identified marker genes in gsBlood Dataset. Top Hub Genes identified in (A) B-cells (B) T-cells (C) Granulocytes of gsBlood dataset are shown as examples. scSCOPE identified markers are indicated by oval shape. Hub genes are ranked based on their level of gene co-expression, indicated by a red-yellow color theme.
(TIF)

S5 Fig scSCOPE reveals potential targets of Tcf7 gene in T-cells. (A) Density Plot showing the relative expression of Tcf7 gene in different cell types of gsBlood dataset. (B) Gene Network Plots for Tcf7 gene show the extensive interactions of Tcf7 gene with genes across multiple pathways in T-cells. In each pathway, Tcf7 gene is placed in the middle with all other genes in the pathway placed in the circumference of the circle. The lines connecting Tcf7 to these genes indicate Pearson’s correlation coefficient, ranging from -1 (red) to +1 (blue), reflecting the strength and direction of correlation. Correlations are separately calculated for two distinct groups: in this case T-cells and all other cell types. Boxplots accompanying the plots contrast the distributions of correlations between these groups. Additionally, p-values from the Kolmogorov-Smirnov test are provided to assess the statistical significance, with the null hypothesis stating that two samples are drawn from the same distribution. (C) Network diagram showing the co-expressed genes of Tcf7 identified by scSCOPE and their annotations based on published experimental results.
(TIF)

S6 Fig scSCOPE can identify genes with low fold change but extensive interactions as marker genes. (A) Genes identified as core and secondary by scSCOPE must pass correlation cutoffs, adjusted differential cutoffs, and differential expression cutoffs to be classified as marker genes. (B) Scatter Plot showing the average log2FC of DEGs identified by Wilcox rank sum method for B-cells in gsBlood dataset. Top marker genes for each cluster are labelled inside a box, indicating their ranking among DEGs from the Wilcoxon analysis. Although Il7r ranks low in terms of average log2FC in B-cells, scSCOPE identifies it as a marker gene in B-cells. (C) The Il7r gene is co-expressed with three core genes identified for B-cells and is also involved in two B-cell pathways. Due to its higher degree of co-expression and involvement in multiple pathways, it ranks higher in the “adjusted differential” metric compared to other genes like Cd74 and Igkc, which have higher fold changes but lower degrees of correlation and pathway involvement.
(TIF)

S7 Fig scSCOPE identifies a higher number of and more stable pathways. (A) Bar plot shows that scSCOPE identifies a higher number of pathways as compared to other methods across all clusters in gsBlood dataset. (B) The stability of each method in identifying pathways in the same cluster across different human PBMC datasets was measured using the procedure highlighted in Fig 2B. scSCOPE identified pathways showed greater stability as compared to pathways identified by other methods. (C) Pathway Network Plots show the difference in expression and co-expression patterns of all the genes within T-cell Receptor Signaling Pathway and Th17 cell differentiation pathway between T-cells and Progenitor Cells. Pathway Network Plots are constructed for both T-cells and progenitor cells, facilitating a comparative analysis of pathway dynamics between the two cell types. In each plot, all the genes in the pathway are placed in the periphery of the circle. Genes are colored as orange (marker genes identified by scSCOPE) or gray. The size of each node corresponds to the average expression of the gene in the group, while edges connecting the nodes represent Pearson’s Correlation between two genes, with thickness indicative of correlation strength. Blue edges signify positive correlations, while red edges indicate negative correlations. (D) Pathway Bar Plot revealing the top pathways identified for T-cells versus progenitor cells, utilizing the novel metric “corrExpress.” Both “posCorrExpress” and “negCorrExpress” are combined to be named as “Final Measure”. This metric integrates differences in both gene `expression and gene-gene co-expression across all genes within the pathway. Pathways depicted with baby pink bars predominantly feature upregulated genes in T-cells, while those with light blue bars denote an abundance of upregulated genes in progenitor cells.
(TIF)

S8 Fig scSCOPE identified more genes within each pathway as compared to other methods. Bar Plots show the number of genes identified by various methods enriched in different pathways across different clusters in gsBlood dataset. The pathways were chosen from the top pathways identified by scSCOPE for each comparison (S3 Table). For every pathway in all clusters, scSCOPE identifies higher number of enriched genes than other methods.
(TIF)

S9 Fig Comparison of scSCOPE with other methods in terms of running time. (A) Runtime of scSCOPE and other DEG methods represented as a line graph with different number of cells. (B) Bar plot showing comparison of SCOPE and scSCOPE to run a single iteration of LASSO algorithm. (C) Comparison of SCOPE and scSCOPE to run a single iteration of co-expression analysis under different number of features (x-axis).
(TIF)

S1 Table Number of marker genes and pathways identified for each cluster by different methods in gsBlood and Human Datasets. (XLSX)

S2 Table Marker Genes Identified for all datasets used in this study by scSCOPE. (XLSX)

S3 Table Pathways identified and ranked for gsBlood and Tabula Muris (B-cells) datasets by scSCOPE. (XLSX)

S4 Table Number of genes identified by scSCOPE and other different methods in different pathways enriched in separate comparisons in the gsBlood dataset. (XLSX)
