# Supplementary figures and images for: Stabilized marker gene identification and functional annotation from single-cell transcriptomic data
Source: PLoS Comput Biol. 2025 Oct 17;21(10):e1013574. doi: 10.1371/journal.pcbi.1013574 (PMC12533881; doi:10.1371/journal.pcbi.1013574)

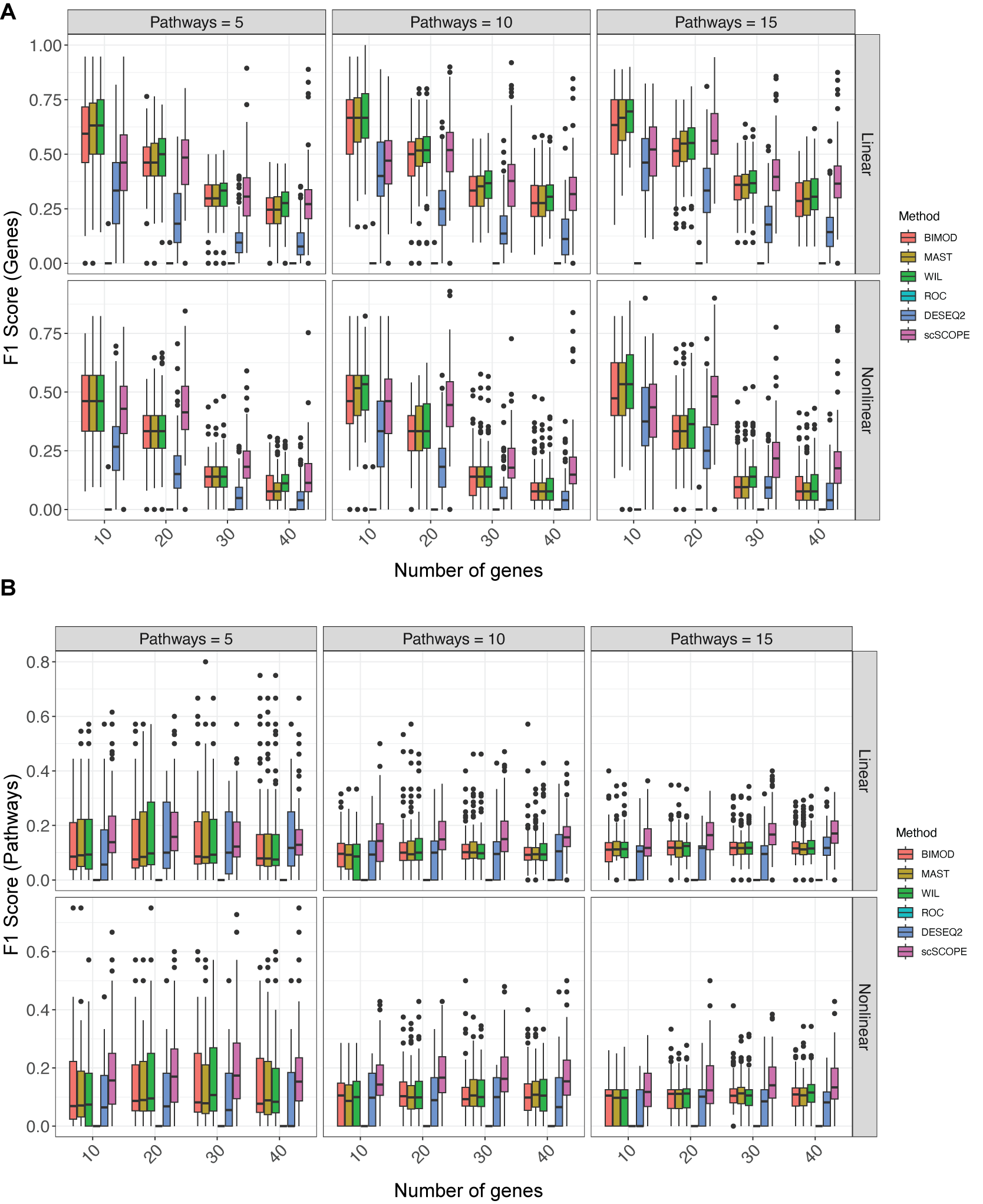

Supplement: S1 Fig — F1 score ={TP/[TP + 0.5 × (FP + FN)]} calculated for the accuracy of scSCOPE and other methods in identifying predictive genes (A) and pathways (B) simulated in the GTEX single-cell gene expression data using linear and non-linear models under different combinations of number of predictive genes and pathways. (TIF) [file pcbi.1013574.s001.tif]

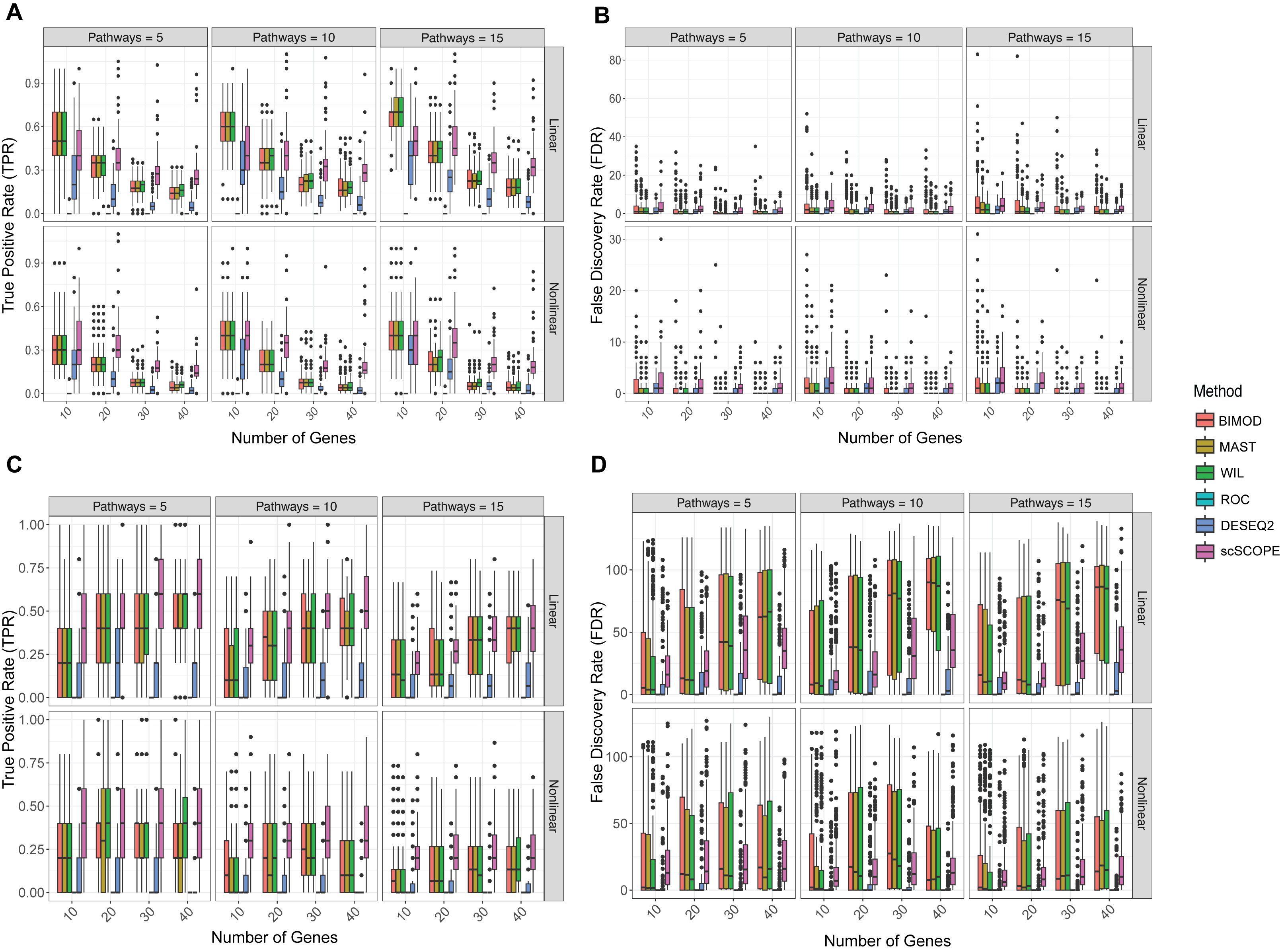

Supplement: S2 Fig — True Positive Rate (TPR) and False Discovery Rate (FDR) calculated for scSCOPE and other methods in identifying predictive genes (A, B) and pathways (C,D) simulated in the GTEX single-cell gene expression data using linear and non-linear models under different combinations of number of predictive genes and pathways respectively. (TIF) [file pcbi.1013574.s002.tif]

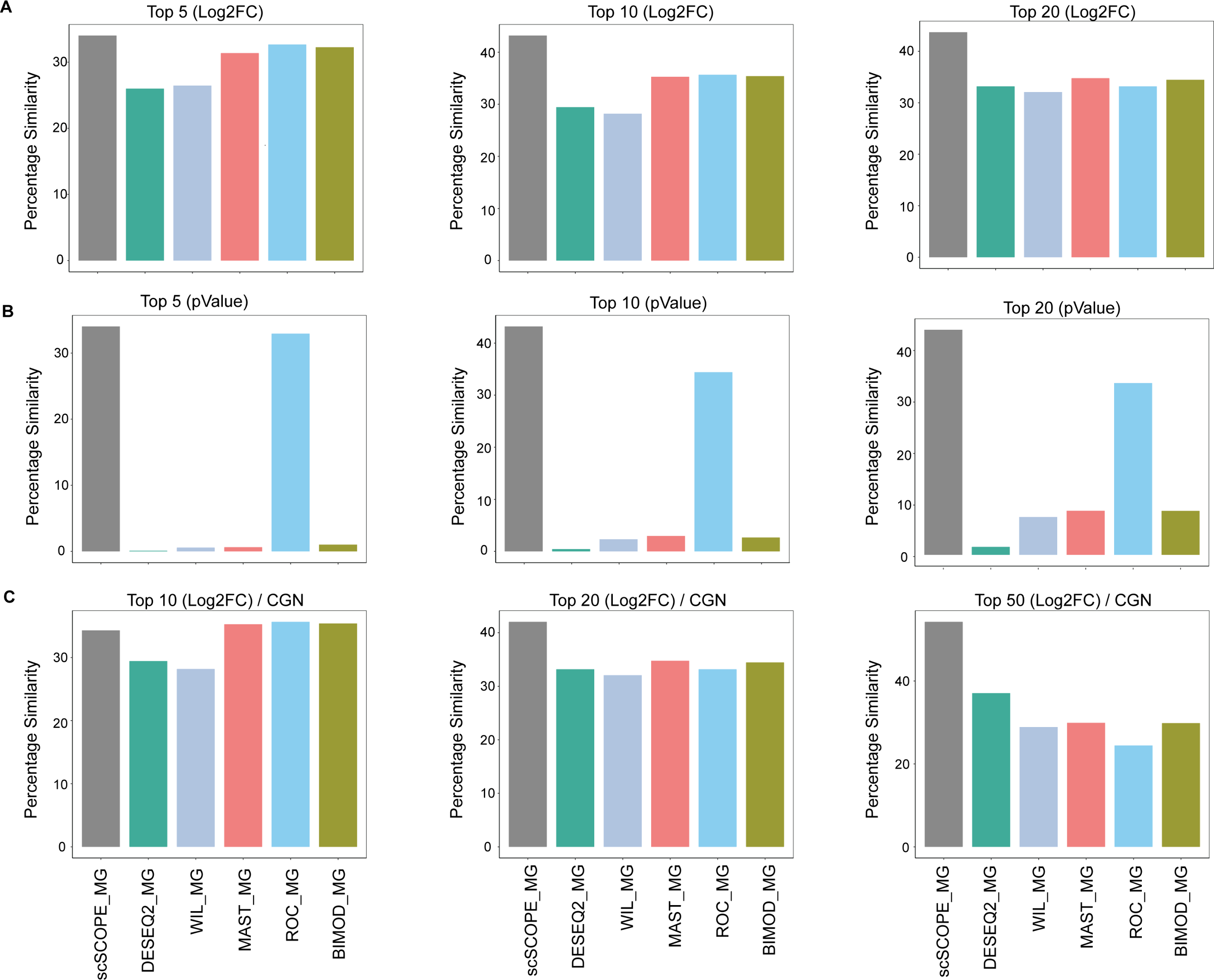

Supplement: S3 Fig — Bar plots illustrating the stability measure of different methods for identifying marker genes in human PBMC datasets [33]. Top DEGs are selected based on their average log fold change in (A), and p-values in (B). (C) Bar plots showing the stability comparison of scSCOPE identified Correlated Gene Network with top genes based on average fold change identified by other methods. (TIF) [file pcbi.1013574.s003.tif]

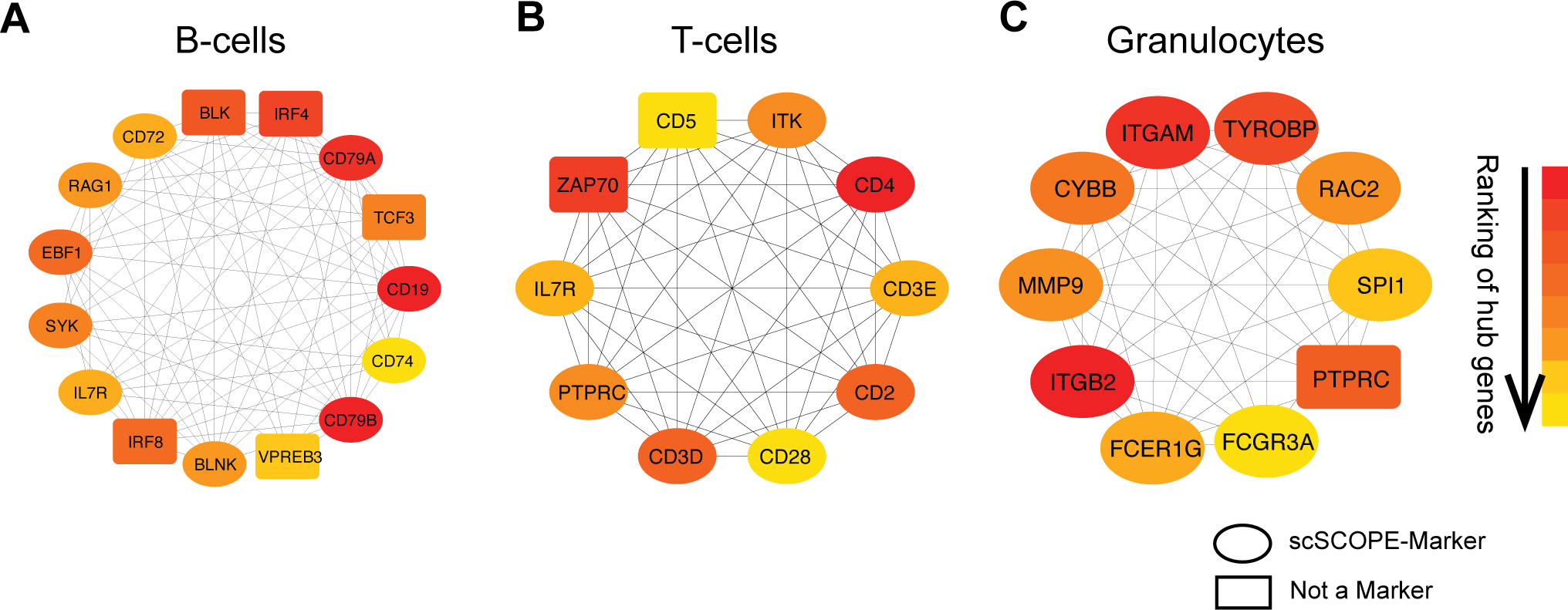

Supplement: S4 Fig — Top Hub Genes identified in (A) B-cells (B) T-cells (C) Granulocytes of gsBlood dataset are shown as examples. scSCOPE identified markers are indicated by oval shape. Hub genes are ranked based on their level of gene co-expression, indicated by a red-yellow color theme. (TIF) [file pcbi.1013574.s004.tif]

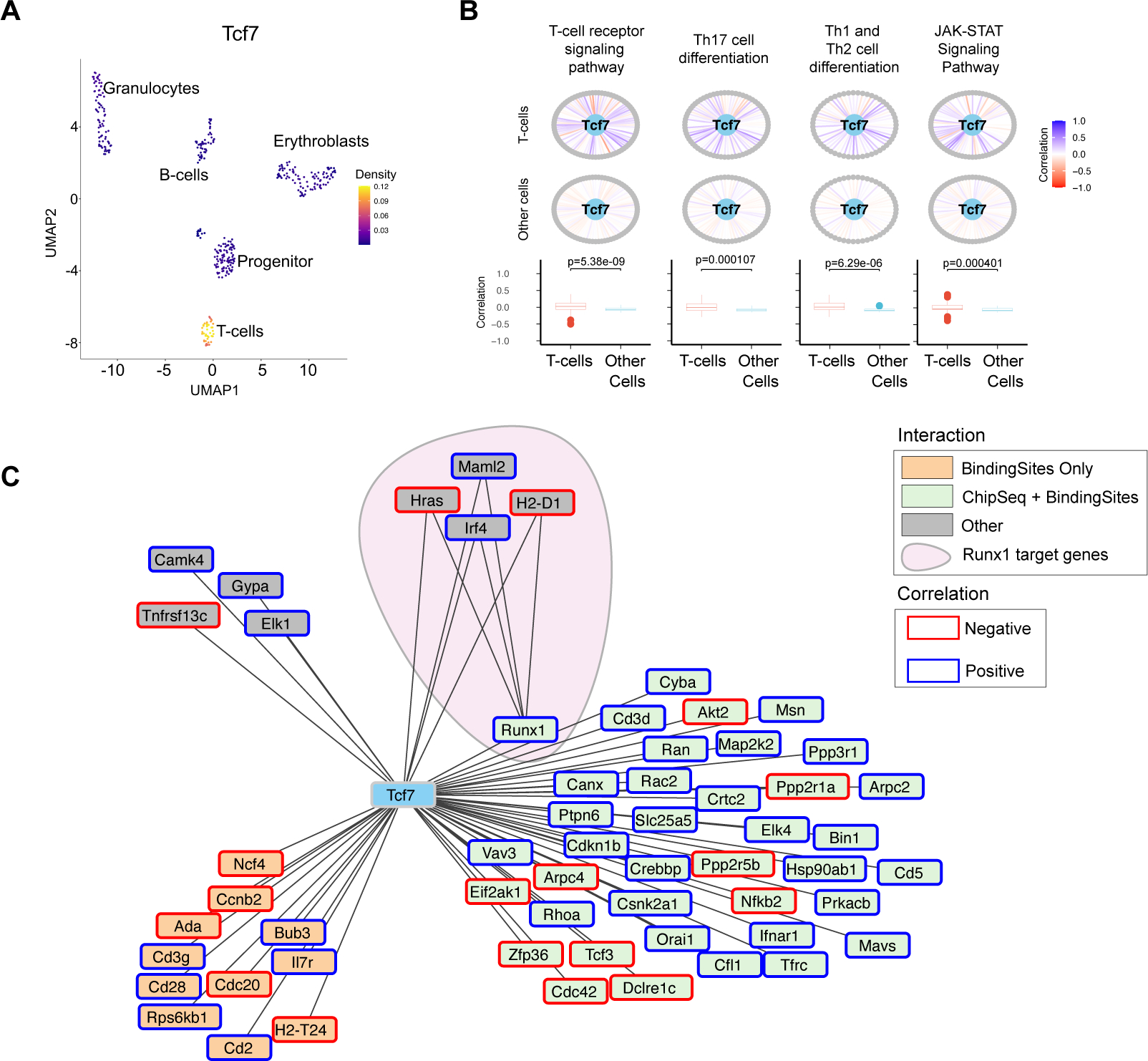

Supplement: S5 Fig — (A) Density Plot showing the relative expression of Tcf7 gene in different cell types of gsBlood dataset. (B) Gene Network Plots for Tcf7 gene show the extensive interactions of Tcf7 gene with genes across multiple pathways in T-cells. In each pathway, Tcf7 gene is placed in the middle with all other genes in the pathway placed in the circumference of the circle. The lines connecting Tcf7 to these genes indicate Pearson’s correlation coefficient, ranging from -1 (red) to +1 (blue), reflecting the strength and direction of correlation. Correlations are separately calculated for two distinct groups: in this case T-cells and all other cell types. Boxplots accompanying the plots contrast the distributions of correlations between these groups. Additionally, p-values from the Kolmogorov-Smirnov test are provided to assess the statistical significance, with the null hypothesis stating that two samples are drawn from the same distribution. (C) Network diagram showing the co-expressed genes of Tcf7 identified by scSCOPE and their annotations based on published experimental results. (TIF) [file pcbi.1013574.s005.tif]

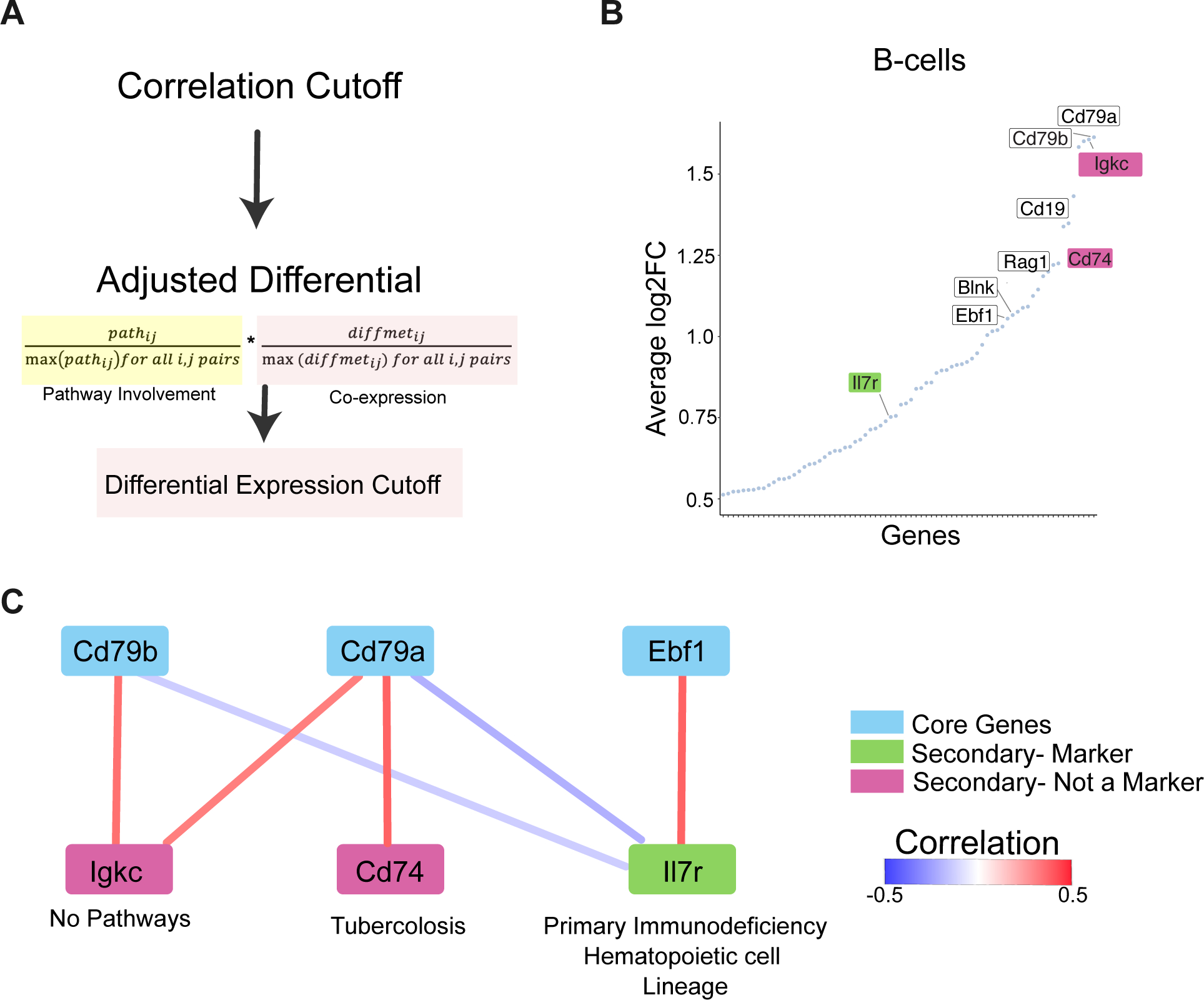

Supplement: S6 Fig — (A) Genes identified as core and secondary by scSCOPE must pass correlation cutoffs, adjusted differential cutoffs, and differential expression cutoffs to be classified as marker genes. (B) Scatter Plot showing the average log2FC of DEGs identified by Wilcox rank sum method for B-cells in gsBlood dataset. Top marker genes for each cluster are labelled inside a box, indicating their ranking among DEGs from the Wilcoxon analysis. Although Il7r ranks low in terms of average log2FC in B-cells, scSCOPE identifies it as a marker gene in B-cells. (C) The Il7r gene is co-expressed with three core genes identified for B-cells and is also involved in two B-cell pathways. Due to its higher degree of co-expression and involvement in multiple pathways, it ranks higher in the “adjusted differential” metric compared to other genes like Cd74 and Igkc, which have higher fold changes but lower degrees of correlation and pathway involvement. (TIF) [file pcbi.1013574.s006.tif]

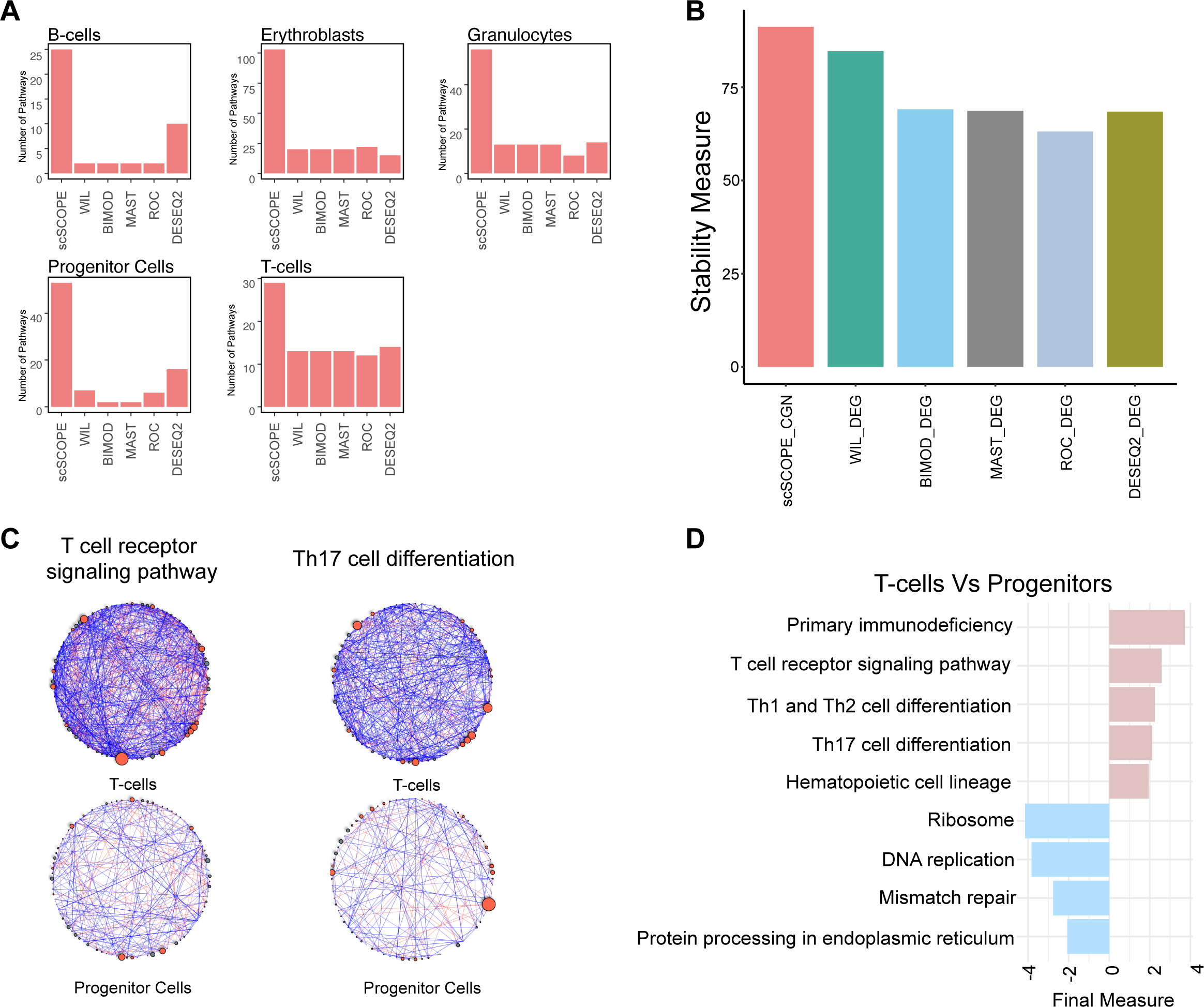

Supplement: S7 Fig — (A) Bar plot shows that scSCOPE identifies a higher number of pathways as compared to other methods across all clusters in gsBlood dataset. (B) The stability of each method in identifying pathways in the same cluster across different human PBMC datasets was measured using the procedure highlighted in Fig 2B. scSCOPE identified pathways showed greater stability as compared to pathways identified by other methods. (C) Pathway Network Plots show the difference in expression and co-expression patterns of all the genes within T-cell Receptor Signaling Pathway and Th17 cell differentiation pathway between T-cells and Progenitor Cells. Pathway Network Plots are constructed for both T-cells and progenitor cells, facilitating a comparative analysis of pathway dynamics between the two cell types. In each plot, all the genes in the pathway are placed in the periphery of the circle. Genes are colored as orange (marker genes identified by scSCOPE) or gray. The size of each node corresponds to the average expression of the gene in the group, while edges connecting the nodes represent Pearson’s Correlation between two genes, with thickness indicative of correlation strength. Blue edges signify positive correlations, while red edges indicate negative correlations. (D) Pathway Bar Plot revealing the top pathways identified for T-cells versus progenitor cells, utilizing the novel metric “corrExpress.” Both “posCorrExpress” and “negCorrExpress” are combined to be named as “Final Measure”. This metric integrates differences in both gene `expression and gene-gene co-expression across all genes within the pathway. Pathways depicted with baby pink bars predominantly feature upregulated genes in T-cells, while those with light blue bars denote an abundance of upregulated genes in progenitor cells. (TIF) [file pcbi.1013574.s007.tif]

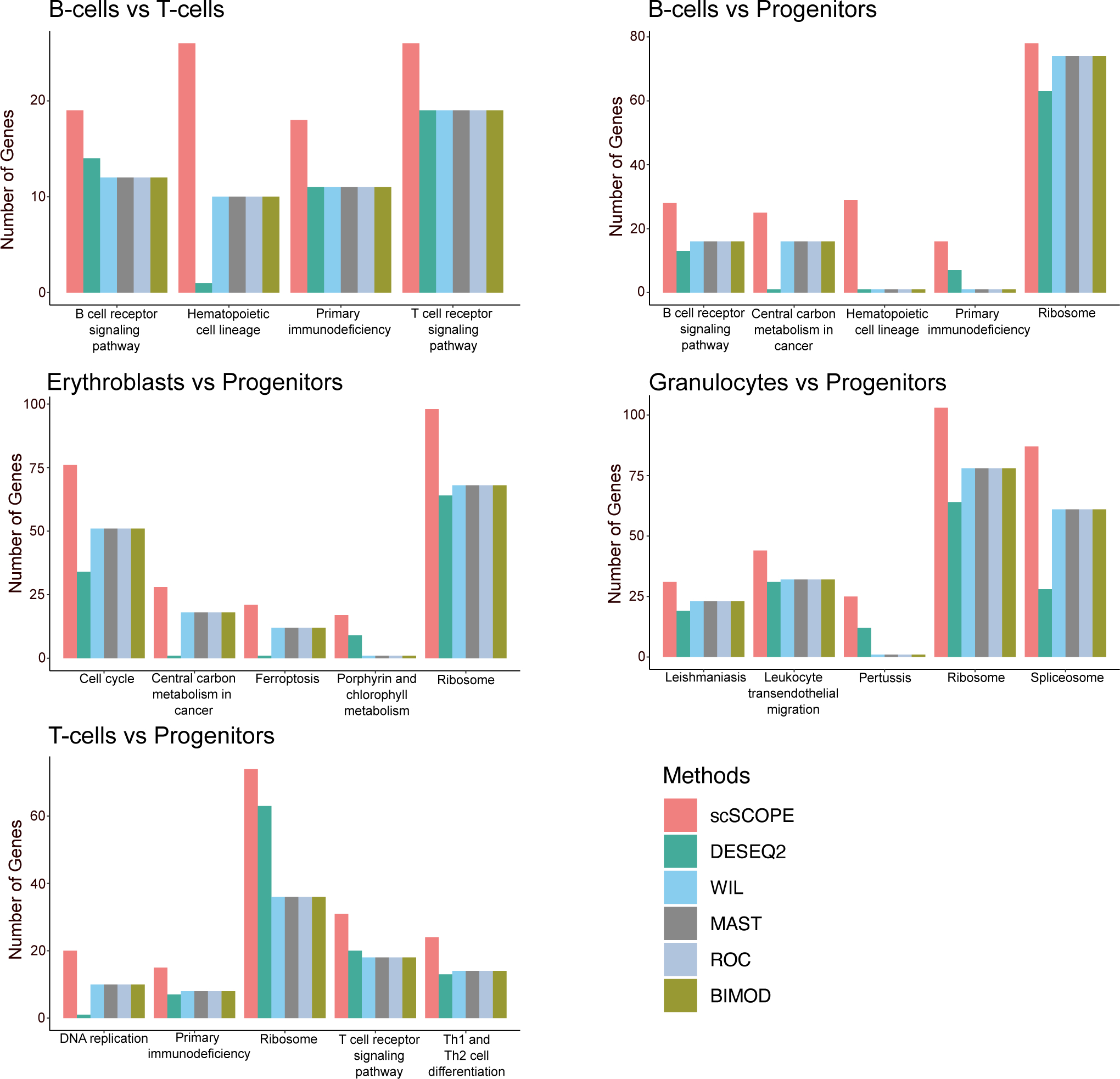

Supplement: S8 Fig — Bar Plots show the number of genes identified by various methods enriched in different pathways across different clusters in gsBlood dataset. The pathways were chosen from the top pathways identified by scSCOPE for each comparison (S3 Table). For every pathway in all clusters, scSCOPE identifies higher number of enriched genes than other methods. (TIF) [file pcbi.1013574.s008.tif]

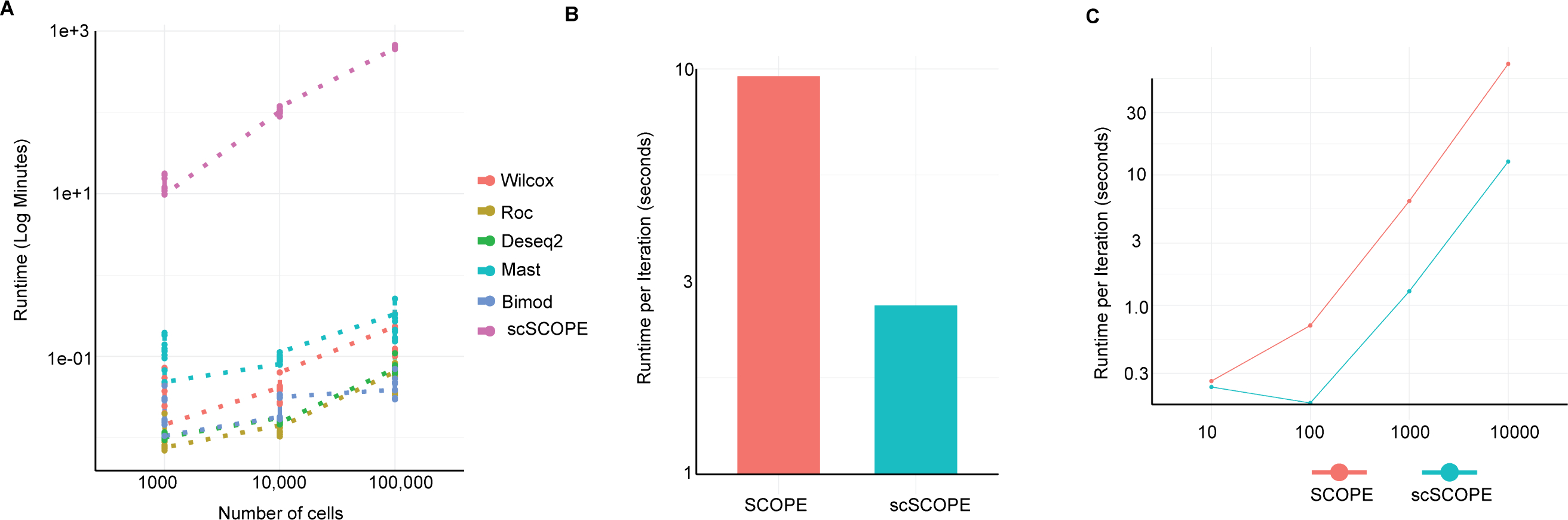

Supplement: S9 Fig — (A) Runtime of scSCOPE and other DEG methods represented as a line graph with different number of cells. (B) Bar plot showing comparison of SCOPE and scSCOPE to run a single iteration of LASSO algorithm. (C) Comparison of SCOPE and scSCOPE to run a single iteration of co-expression analysis under different number of features (x-axis). (TIF) [file pcbi.1013574.s009.tif]
